# Early‐life exercise effects on Achilles tendon in mice selectively bred for high voluntary wheel‐running behavior

**DOI:** 10.14814/phy2.70515

**Published:** 2025-08-19

**Authors:** Miles Valencia, Jenna Monroy, Theodore Garland, Angela M. Horner

**Affiliations:** ^1^ Department of Biology California State University San Bernardino California USA; ^2^ Department of Natural Sciences Claremont Colleges Claremont California USA; ^3^ Department of Evolution, Ecology, and Organismal Biology University of California Riverside California USA; ^4^ Present address: School of Biological Sciences Biology University of California Irvine California USA

**Keywords:** artificial selection, biomechanics, locomotion, maturation, tendon, wheel running

## Abstract

Exercise increases muscle and bone strength and mass, but effects on tendons are less documented. We investigated the impact of voluntary exercise (wheel running) during early‐life exercise (weanling; 3 weeks old) compared to post‐skeletal maturity (young adult; 9 weeks old) on tendon morphology and material properties. We utilized a selectively bred High Runner (HR, *N* = 40) mouse line and a control line (*N* = 40). Mice underwent 8 weeks in cages either with or without wheels. HR mice ran ~3‐fold more and were smaller than controls, but exercise reduced body mass in both lines. Tendon cross‐sectional area was unaffected, but tendon length showed a line*exercise interaction (*p* = 0.0410) and a near‐significant line*age interaction (*p* = 0.0866). HR mice broadly had greater yield stress (*p* = 0.0262) and tended toward higher failure stress (*p* = 0.0676) than controls. Work to failure was greater in younger cohort mice (*p* = 0.0435), and marginal age‐related interactions were observed for modulus (line*exercise, *p* = 0.0632) and yield strain (line*age, *p* = 0.0535). HR mice were more responsive to exercise; older exercised HR mice had shorter tendons (*p* = 0.0282), and younger exercised HR mice showed lower yield and failure strains than sedentary counterparts (*p* = 0.0445, 0.0246). Exercise and its relative timing produced slight but complex effects on tendon properties, with HR mice showing the strongest structural and mechanical responses.

## 1 INTRODUCTION

Tendons are highly specialized tissues that connect muscle to bone in vertebrates. In addition to providing an attachment point for muscles, tendons can also enhance locomotor performance by resisting high tensile loads, amplifying muscle power, conserving energy, or dissipating energy (Roberts & Azizi, 2011). Structural changes induced by loading can alter tendon cross‐sectional area (CSA), the angle and density of collagen fibrils, and advanced glycation end products (AGEs), all of which contribute to tendon mechanical properties such as stiffness/modulus and failure strength (Barin et al., 2017; Narici & Maganaris, 2007; Slane et al., 2015). Thus, changes to tendon morphology and composition have the potential to greatly impact musculoskeletal functionality, mobility, and stability.

Altered loading during exercise training can change the structure, composition, and mechanical properties of tendon, potentially increasing its excess capacity or safety factor. In humans, endurance running can improve tendon performance directly by hypertrophy; for example, tendons of long‐distance runners exhibited cross‐sectional areas (CSA) ~30% greater than non‐runners (Heinemeier & Kjaer, 2011). However, tendon mechanical properties (e.g., stiffness/modulus, failure strength) do not strictly correlate with morphology; thus, tendon hypertrophy does not always translate directly to increased stiffness (LaCroix et al., 2013). In addition, the differences in intensity and frequency utilized in training protocols yield varied results in tendon morphology and mechanical properties (Buchanan & Marsh, 2001; de Cássia Marqueti et al., 2017; Heinemeier et al., 2012; Klitgaard, 1988; Legerlotz et al., 2007; Simonsen et al., 1995; Wood & Brooks, 2016). Among a wide diversity of vertebrates that includes humans, rats, pigs, chickens, mice, and rabbits, researchers have found that training leads to an increase in tendon stiffness (Arampatzis et al., 2010; Buchanan & Marsh, 2001; Couppé et al., 2008; Heinemeier et al., 2012; Kubo et al., 2006) and strength (Nakagaki et al., 2007; Woo et al., 1981), although several studies in the same taxa have demonstrated no effect (rats, Inhofe et al., 1995; humans, Rosager et al., 2002; rats, Huang et al., 2004; humans, Karamanidis & Arampatzis, 2006). More specifically, both resistance training and uphill running have been effective at increasing tendon strength in rats and humans (Heinemeier et al., 2012; Kaux et al., 2013), but endurance training has resulted in less conclusive outcomes in the same species (Karamanidis & Arampatzis, 2006; Milgrom et al., 2014). Studies disagree on the mechanisms responsible for tendon plasticity, as well as the net mechanical effect of exercise. In addition to changes in gross CSA, exercise can induce alterations in tendon AGE content, proteoglycan concentration, and matrix turnover rate, but the net mechanical effects of these changes remain unclear (Svensson et al., 2017).

Tendon composition and structure changes over ontogeny, particularly during the window of time between postnatal growth and skeletal maturity. As tendons mature, cell density significantly decreases, largely due to the expansion of the extracellular matrix (Ippolito et al., 1980). Maturation also affects cell morphology: young, immature tendons contain relatively round cells, which transition to long, spindle‐shaped cells with less cytoplasm and reduced synthetic activity as the tendon matures (Lavagnino et al., 2013; Svensson et al., 2017). Decreased cell synthesis and changes in tendon cell morphology among mammals seem to be associated with maturation, but not with aging (reviewed in Svensson et al., 2017; Thorpe et al., 2017). Previous studies in rats (Almekinders & Deol, 1999) and horses (Patterson‐Kane et al., 1997) show higher collagen synthesis in immature tendons compared to mature ones, indicating that the observed decline in synthetic activity is associated more with maturation than aging. The fact that cell numbers and synthetic activity peak during postnatal growth and slow after maturity is further supported in studies on humans (Heinemeier et al., 2013), horses (Smith et al., 2002), and mice (Gumucio et al., 2014).

The extent to which tendon may remodel to loading in vertebrates is limited by the fact that cell synthesis activity tends to peak at sexual maturation and subsequently declines (Connizzo et al., 2013). Thus, the core matrix composition of a tendon remains relatively unchanged post‐adolescence (Heinemeier et al., 2013). Tendon hypertrophy, such as in response to mechanical loading, has been shown to similarly be limited to the superficial‐most regions of the tendon, designated as the neotenon. This phenomenon of the tendon core remaining unchanged after growth has been documented in several studies (Gumucio et al., 2014; Heinemeier et al., 2013; Kubo et al., 2014), but the implications for later‐life resistance to tendinopathies are largely unexplored (but see Lenskjold et al., 2015). In equine studies, evidence from yearling horses suggests that there may be a “window of opportunity” to ideally load young tendon for maximum later‐life tendon health (Smith et al., 2002).

In this study, we sought to investigate the isolated effects of early‐life loading on mature, but not aged, mouse tendon. We exposed mice to voluntary wheel exercise for 8 weeks immediately following weaning (aged 3 weeks) and compared tendon morphology and material properties to a cohort of mice with exercise exposure beginning after sexual maturity, from 9 weeks of age through 17 weeks. To further explore the role of loading frequency and intensity on tendon loading, we utilized a High Runner (HR) mouse line from an ongoing artificial selection experiment in which four replicate lines have been bred for voluntary wheel‐running behavior for >100 generations while four nonselected lines serve as controls (Careau et al., 2013; Swallow et al., 1998; Wallace & Garland, 2016). Use of an HR line should increase the statistical power to detect training effects from voluntary wheel running, if they occur, as HR mice regularly outrun control line mice approximately threefold in daily distance (e.g., Meek et al., 2014). We hypothesized that tendons from younger, immature mice would exhibit greater plasticity in response to exercise than tendons from older mice near skeletal maturity, including greater changes to failure stress and modulus. If the intensity and frequency of tendon loading impact tendon remodeling, then HR mice should exhibit a greater magnitude of response to loading. However, HR mice already tend to have longer and thinner tendons (Castro et al., 2024), and thus HR tendon may be preadapted for running.

## 2 METHODS

### 2.1 Animals

Mice were obtained from the High Runner (HR) selection experiment for high voluntary wheel‐running behavior, which has been described in detail elsewhere (Swallow et al., 1998). Briefly, 224 Hsd:ICR mice (*Mus domesticus*) were used as progenitors for eight closed lines. Within four control lines, mice are bred without regard to how much they run on wheels. Within the four HR lines, young adult mice are given access to Wahman‐type activity wheels (1.12 m circumference; Lafayette Instruments) for 6 days. Based on the fifth and sixth days of testing, males and females with the highest total wheel revolutions for their family are selected to reproduce (within‐family selection). For both control and HR lines, mice breed only within their line, and siblings are not allowed to mate.

**FIGURE 1:**
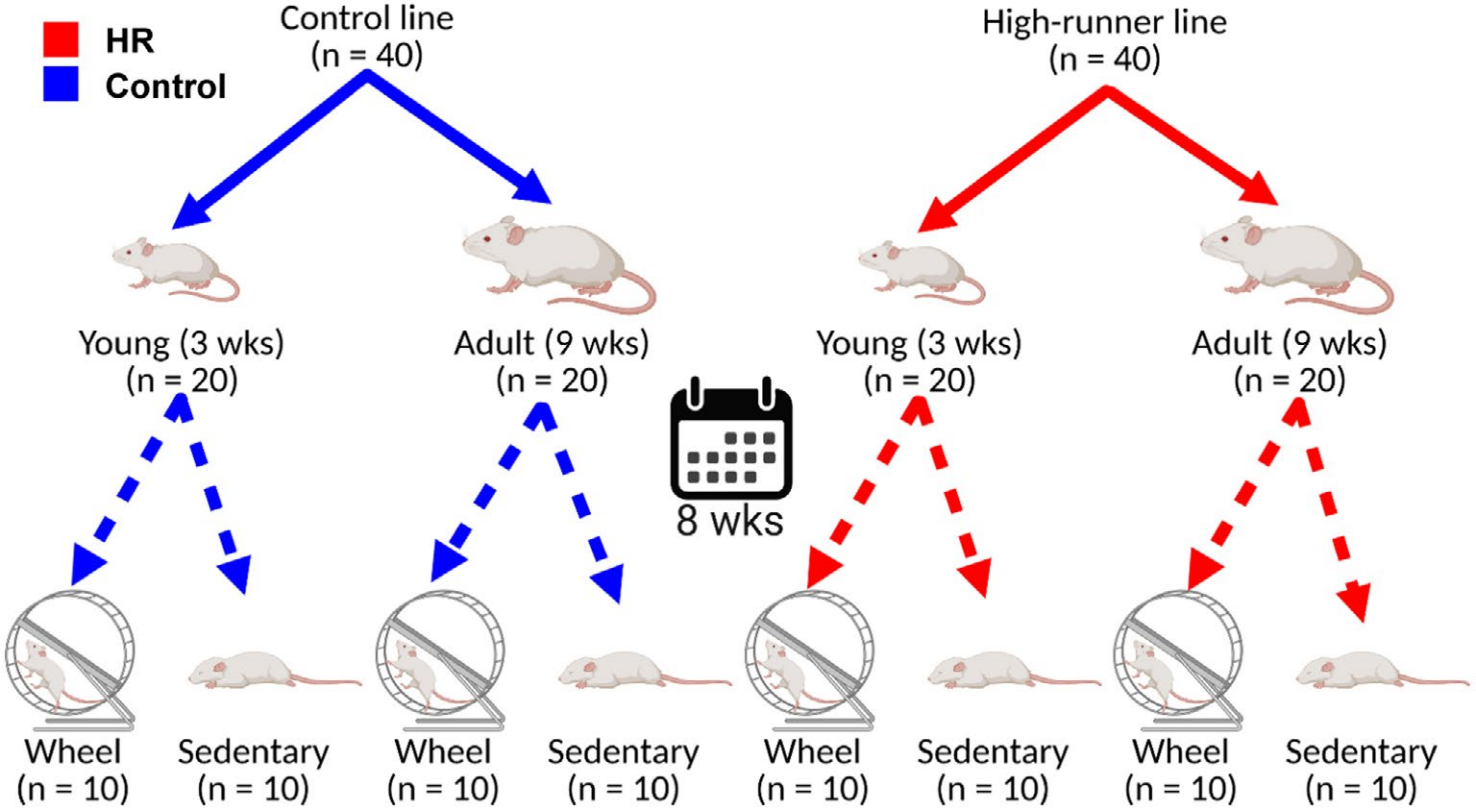
Experimental design and sample sizes for two lines of mice (HR and control) exposed to exercise at either weanling (3 weeks old) or young adult (9 weeks old) and compared to sedentary mice. Mice from control and high‐runner lines (normal‐/high‐activity) were separated into 2 age cohorts including young (3 weeks) and adult (9 weeks) groups. These cohorts were then separated into two exercise groups (sedentary/wheel) for 8 weeks. Illustration was created with BioRender.com.

For the present study, a total of 80 female mice from generations 92 and 95 were weaned at 21 days of age and housed in cages specific to exercise treatment. Mice were categorized by line, age, and exercise treatment (Figure 1); 40 mice in total were used from one HR line 7 (lab designation) and 40 mice from control line 1. Each line was separated into two age cohorts (*n* = 20) corresponding to before (young, 3 weeks; Gen 92) and after (adult, 9 weeks, Gen 95) the beginning of skeletal maturity (Farooq et al., 2017). Ten mice in each age cohort were housed individually in cages with activity wheels (9.2 cm circumference; Columbus Instruments Mouse Home Cage Running Wheel) that recorded revolutions run in 30‐min intervals for 8 weeks. Sedentary mice (10 mice/age cohort) were kept in standard housing (33.7 cm long × 18.2 cm deep × 14 cm wide). Following IACUC protocol (19‐020) at CSUSB and veterinarian approval, all mice were given food (LabDiet Standard Rodent Diet) and water ad libitum; room temperature was kept at (21.5°C) and photoperiod at 12 h:12 h, with lights on at 07:00 h.

After 8 weeks, all mice were euthanized and stored at −20°C for dissection and materials testing at a later date. Tendons stored at −20°C have been reported to not have significantly different mechanical properties compared to freshly dissected tendon (Goh et al., 2010); nor do the mechanical properties significantly change after being stored at −20°C for up to 360 days (Ng & Chou, 2003). To further ensure mechanical properties were not altered, tendons remained frozen in situ within the limbs until mechanically tested (Chen et al., 2011).

### 2.2 Morphometrics

**FIGURE 2:**
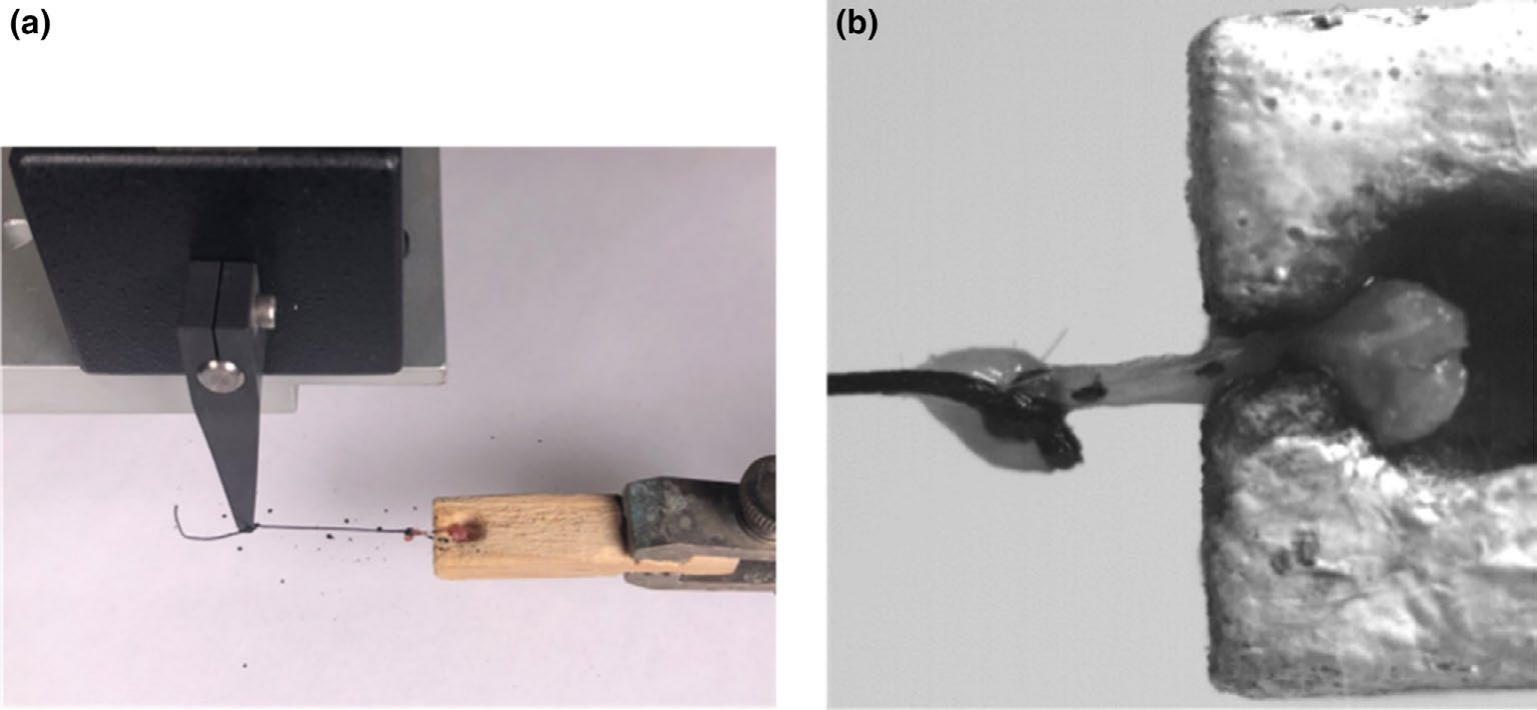
Tendon testing apparatus with custom‐made wooden rig to hold tendon without direct clamping. (a) With the Achilles tendon insertion intact, the calcaneus was wedged within the conical slot of the wooden rig (see Section 2). The proximal tendon was bound by a silk thread that was attached to the ergometer. (b) Two dots of India ink are visible; the most distal dot was used to calculate tendon length change. The suture knot was tied distally to the muscle‐tendon junction to estimate the proximal end of the tendon.

The Achilles tendon (combined tendons of gastrocnemii and soleus) was carefully dissected from the hindlimb to take morphometric measurements. The Achilles tendon of the right hindlimb was separated from the surrounding connective tissue but kept intact and moist with phosphate‐buffered solution throughout imaging and testing. Images were recorded via two methodologies: (1) the tendon was positioned in front of a mirror angled at 45° so that a digital SLR camera (Canon EOS Rebel T3) captured images from dorsal and lateral views; or 2) the tendon was positioned within the mechanical testing rig (Figure 2). ImageJ (NIH) was used to measure the dorsal and lateral diameters (d1, d2) as well as the tendon length. We assumed that the tendon is elliptical in cross‐section and calculated the cross‐sectional area (Equation 1).

(1) $$\mathrm{CSA}=\;\frac{1}{2}d_{1}*\frac{1}{2}d_{2}*\pi$$

The right Achilles tendon was used for mechanical testing, and the left plantarflexor muscles were weighed to assess whether changes in tendon morphometrics corresponded to muscle hypertrophy. We did not compare morphologies or properties between right and left hindlimbs, but previous research in HR lines was indicative of decreasing asymmetry between limbs (Garland Jr. & Freeman, 2005).

### 2.3 Mechanical testing

To secure the position of the Achilles tendon during mechanical testing, two regions were kept intact: a portion of the calcaneus where the Achilles tendon inserts and the muscle‐tendon junction where the tendon becomes aponeurosis. We modified the experimental setup described by Probst et al. (2000). A wooden block with a conical slot held the calcaneus in place, thus increasing the total measurable distance of the distal region of the tendon compared to setups that use suture to tie off the tendon (Figure 2). The proximal region distal to the muscle‐tendon junction was bound with a 2–0 silk surgical knot and reinforced with VetBond to prevent slippage (Komatsu et al., 2007). The silk thread was attached to a dual‐mode ergometer (305C‐LR, 10.0 N, Aurora Scientific Inc., Ontario, Canada) for ex vivo materials testing. The ergometer was fixed to a three‐axis manipulator that adjusted the initial length of the tendon. The time and force measurements from the ergometer were recorded at 1000 Hz using a 16‐bit A/D converter (National Instruments, TX, USA) and a LabChart 8 software program (LabChart 8 v. 8; AD Instruments). We used visual tracking to calculate tendon deformation because compliant structures (e.g., suture) in series with the tendon may inflate length changes recorded by an ergometer. We used a 10/0 detail paintbrush to apply two dots of India ink: distally near the calcaneal insertion and proximally near the suture knot (Figure 2). A high‐speed camera (XC‐2, XCitex) equipped with a macro lens (Sigma 105 mm F2.8 EX DG OS HSM) recorded the dorsal view of the tendon at 300 fps using ProCapture software (ProCapture, XCitex). All data were synchronized using an external trigger. The India ink dots were tracked using ProAnalyst software program (ProAnalyst, XCitex) and then exported as x‐y coordinates for data analysis.

We used cyclic loading and ramp‐to‐failure tests to measure tendon mechanical properties. Prior to testing, the tendon was extended until under mild tension to approximate in vivo resting length (L_0_). A preconditioning protocol of three cyclic loads was applied over 2.5 s to realign collagen fibers along the long axis of the tendon (Schatzmann et al., 1998). The ramp‐to‐failure test applied 550 g over 2.5 s to measure the force and deformation of a tendon to rupture. Tendon is relatively impervious to temperature (Huang et al., 2004; Rigby et al., 1959), but all mechanical tests were nevertheless conducted at room temperature (23°C).

### 2.4 Data analysis

Tendon mechanical properties were calculated as follows: length change (deformation) was measured by calculating the distance between the x, y positional data from proximal and distal India ink dots in Igor Pro Software (Wavemetrics Inc., OR, USA). The resting length (L_0_) was measured while the tendon was under resting tension prior to the second or third cyclic load. A smoothing spline was applied to the strain data from cyclic loading tests before measuring L_0_ and deformation. Stiffness was calculated by graphing a force‐length curve and measuring the slope of the linear region (Equation 2). Morphometric measurements, including cross‐sectional area (CSA) and resting length (L_0_) were used to convert stiffness to elastic modulus (Equation 3). During ramp‐to‐failure tests, yield force and strain were recorded at the length at which the slope of the linear region began to decrease. Peak forces experienced during ramp‐to‐failure tests were recorded as the failure force and converted to stress (Equation 4); the strain at the peak force value was recorded as failure strain. Work to failure was calculated as the area under the force‐deformation curve until failure.

(2) $$\text{Stiffness}=\frac{\text{Force}\;\left(g\right)}{\text{ΔLength}}$$

(3) $$\text{Elastic Modulus}=\frac{\text{Stress}\;\left(g*{\mathrm{cm}}^{-2}\right)}{\text{Strain}}$$

(4) $$\text{Failure Stress}=\frac{\text{Peak Force}\;\left(g\right)}{\mathrm{CSA}\;\left({\mathrm{cm}}^{2}\right)}$$

### 2.5 Statistical analyses

Statistical analyses were performed using SAS Procedure Mixed. We conducted a linear model where line (control, high‐runner), age (during training exposure; young, adult), and training (sedentary, wheel access) were treated as main effects, and body mass as a covariate. All 2‐way and the 3‐way interactions of main effects were also included in the model. All traits were log_10_‐transformed prior to analyses, and statistical significance was determined at *p* ≤ 0.05. To correct for performing multiple statistical tests on related data, we used the 70 *p* values for main effects, their interactions, and body mass as a covariate reported in Table 1 and calculated the Bonferroni correction to control the Familywise Error Rate at 5% (a very conservative approach). To further investigate the potential impact of extreme exercise, we also compared differences in least‐squares means of mice from the HR line only.

**TABLE 1:** Statistical results for morphometrics and tendon mechanical properties.

Summary of *p* values	*N*	Age	Line	Exercise	Line* exercise	Line*age	Exercise*age	Line *exercise*age	L10BodyMassg	Difs. of LS means	
Trait										Young HR sed vs. ex	Older HR sed vs. ex
Log10 (Body mass, g)	79	**0.0004**	**0.0032**	**0.0034**	0.6219	0.4355	0.6232	0.1205	NA	**0.0152**	0.3486
Log10 (Gastrocnemius mass, g)	31	*0.0765*	**0.0261**	*0.0851*	0.5017	0.7130	0.2519	NA	0.1877	NA	NA
Log10 (Cross‐sectional area, mm^2^)	73	0.2487	0.3454	0.6701	0.5407	0.4803	0.3117	0.5872	0.6018	0.5417	0.6840
Log10 (Tendon length, mm)	73	0.4203	0.2028	*0.0859*	**0.0410**	*0.0866*	0.9746	0.1963	0.5507	0.1162	**0.0282**
Log10 (Yield stress, g/ mm^2^)	73	0.1910	**0.0262**	0.9856	0.6034	0.6777	0.4236	0.8102	0.2113	0.9143	0.6950
Log10 (Yield strain)	71	0.8270	0.6279	0.2962	0.4309	*0.0535*	0.3309	*0.0640*	0.5905	**0.0445**	0.7623
Log10 (Failure stress, Pa)	73	0.2779	*0.0676*	0.9317	0.8083	0.4455	0.2980	0.6915	0.2446	0.7289	0.8183
Log10 (Failure strain)	71	0.1507	0.1271	0.1697	0.2244	0.2201	0.2910	0.2627	0.9000	**0.0246**	0.8300
Log10 (Modulus)	72	0.4546	0.7100	0.9693	0.4655	0.7368	*0.0632*	0.6808	0.7575	0.3792	*0.0794*
Log10 (Work to failure, N‐m)	69	**0.0435**	0.1768	0.2665	0.3986	0.5168	0.6850	0.5429	0.3071	0.1399	0.7123

*Note*: Results from a mixed model analysis with age, line, and exercises as main effects and body mass as a covariate. All traits were log_10_‐transformed prior to analyses. All values where *P* values are bolded indicate *p* < 0.05 and those in italics are *p* < 0.10, with no adjustment for multiple comparisons (see Section 2). NA indicates could not be included in model due to small sample size.

Before analyzing the data, several mice were excluded due to problems recording wheel running. In addition, nine mice were excluded due to complications and errors in materials testing, such as VetBond covering the tendon or the proximal knot unraveling before tendon rupture. Finally, a combination of subjective and objective tests (e.g., standardized residuals >3) was used to identify and remove outliers to meet assumptions of normality and homoscedasticity in the residuals from the statistical models.

## 3 RESULTS

### 3.1 Voluntary wheel running

**FIGURE 3:**
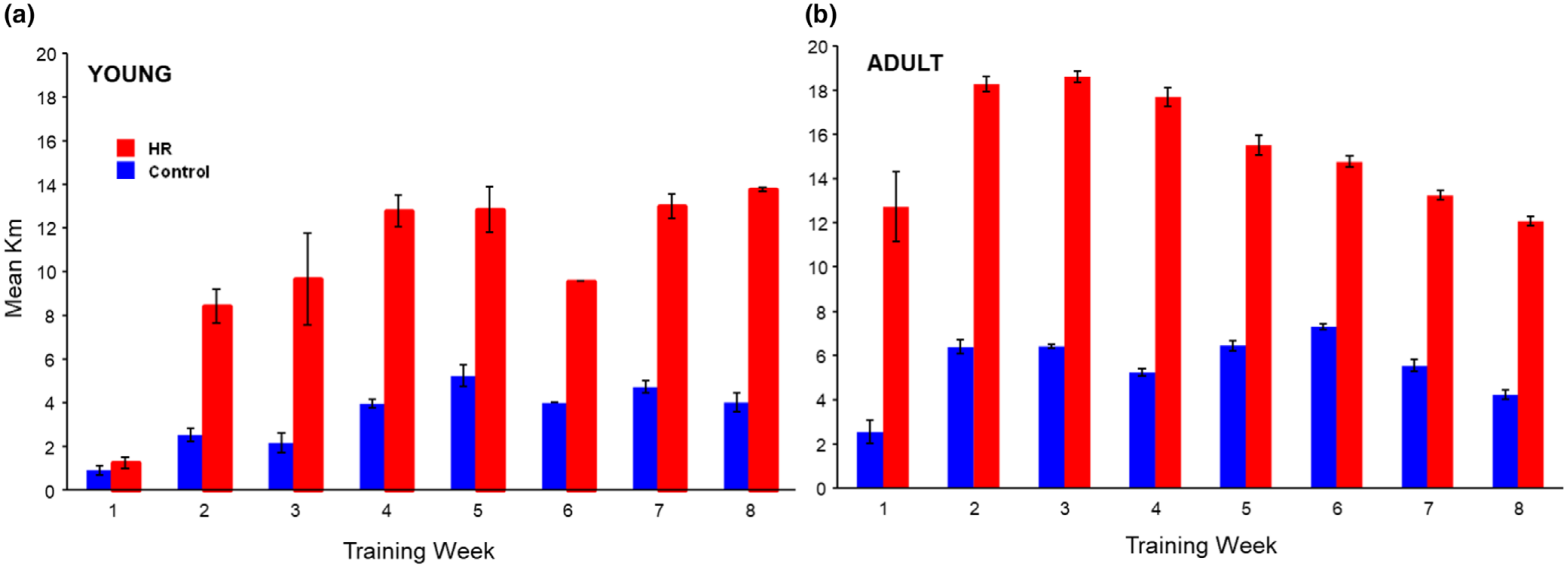
Pooled average weekly wheel running for young (left) and young adult (right) HR (red) and control (blue) mice during 8‐week training. Mean distance run per each training week for young cohort (3‐week‐old training start; a, open bars) and young adult (9‐week‐old training start; b, filled bars) mice from Control (blue) and HR (red) lines. Error bars are ±SEM.

As expected, based on many previous studies (e.g., see Girard et al., 2002; Meek et al., 2009), mice from the HR line consistently showed an approximately 3‐fold difference in distance run compared to the control line (Figure 3).

### 3.2 Significance levels

Although formal multiple comparisons corrections such as the Bonferroni correction and the positive False Discovery Rate (Q‐value) approach suggest that nearly all the significant values from the >70 *p* values in Table 1 are lost (assuming *p* = 0.05), we are interpreting any result with a *p* value <0.10 as having some biological relevance. Additionally, we further investigated between‐cohort effects by examining differences of Least Squares Means results from the mixed model analyses. Below we will discuss those results further.

### 3.3 Morphometrics

**FIGURE 4:**
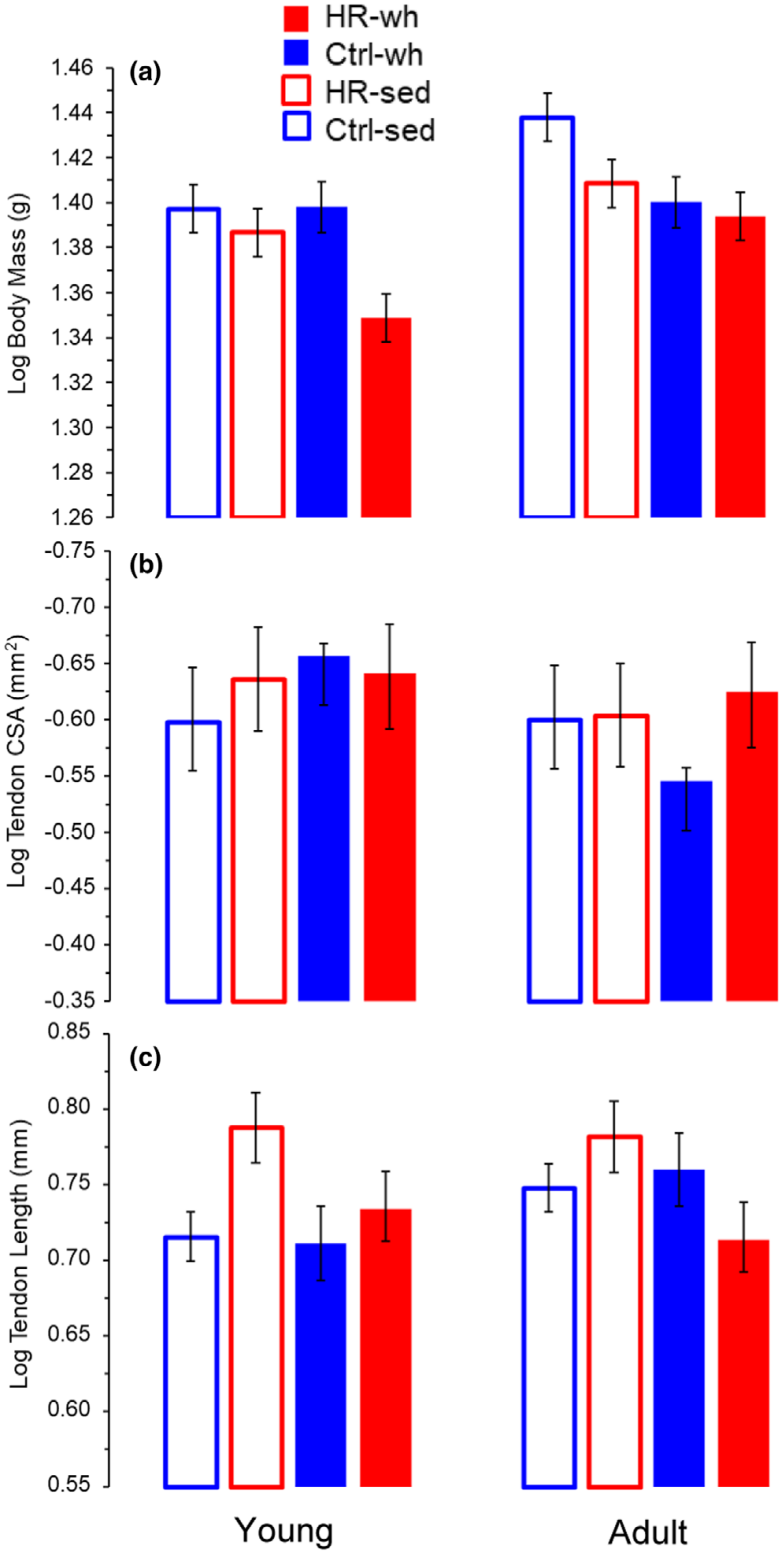
Least squares means (LSM) for morphological measures taken from HR and Control mice after the training period beginning as either young (3 weeks old, left side of graphs) or young adult (9 weeks old, right side of graphs). Least squares means (LSM) and standard errors of (a) body mass, (b) gastrocnemius muscle mass, (c) tendon cross‐sectional area, and (d) tendon length from mixed model analyses with Age, Exercise, Line as fixed effects, and body mass as a covariate where appropriate. Control mice are indicated in blue, HR mice in red, and wheel exercised mice are indicated with a filled bar, whereas sedentary cohorts have bars with no fill. Tendon length (d) was significantly affected by the interaction of linetype*exercise (*p* = 0.0410) such that sedentary HR mice had longer tendons than control line mice and HR wheel access mice. Corresponding statistical analyses are listed within Table 1.

Considering unadjusted *p* values, log‐transformed body mass was significantly affected by line, exercise, and age (Figure 4; Table 1). When averaged across all subgroups, control mice were heavier than HR mice (differences of least squares means *p* = 0.0032); mice not given wheel access were heavier than the active group (*p* = 0.0034); and those who began wheel access at 9 weeks of age were heavier than those beginning exercise at 3 weeks (*p* = 0.0004). With body mass as a covariate (*p* = 0.1877), combined medial and lateral gastrocnemius muscle mass was larger in control mice than in HR mice (*p* = 0.0261); the adult cohort averaged larger than the young cohort (*p* = 0.0765); and exercise tended to decrease muscle mass (*p* = 0.0851), with no interaction effects (Table 1). The cross‐sectional area of the Achilles tendon was not significantly affected by body mass, line, age, exercise, or interactions of the main effects (Figure 4; Table 1). However, exercise and line had a significant interaction effect on tendon length (mm, log‐transformed; *p* = 0.0410; Figure 4; Table 1) such that HR sedentary mice had longer tendons than HR wheel access mice.

### 3.4 Mechanical testing

**FIGURE 5:**
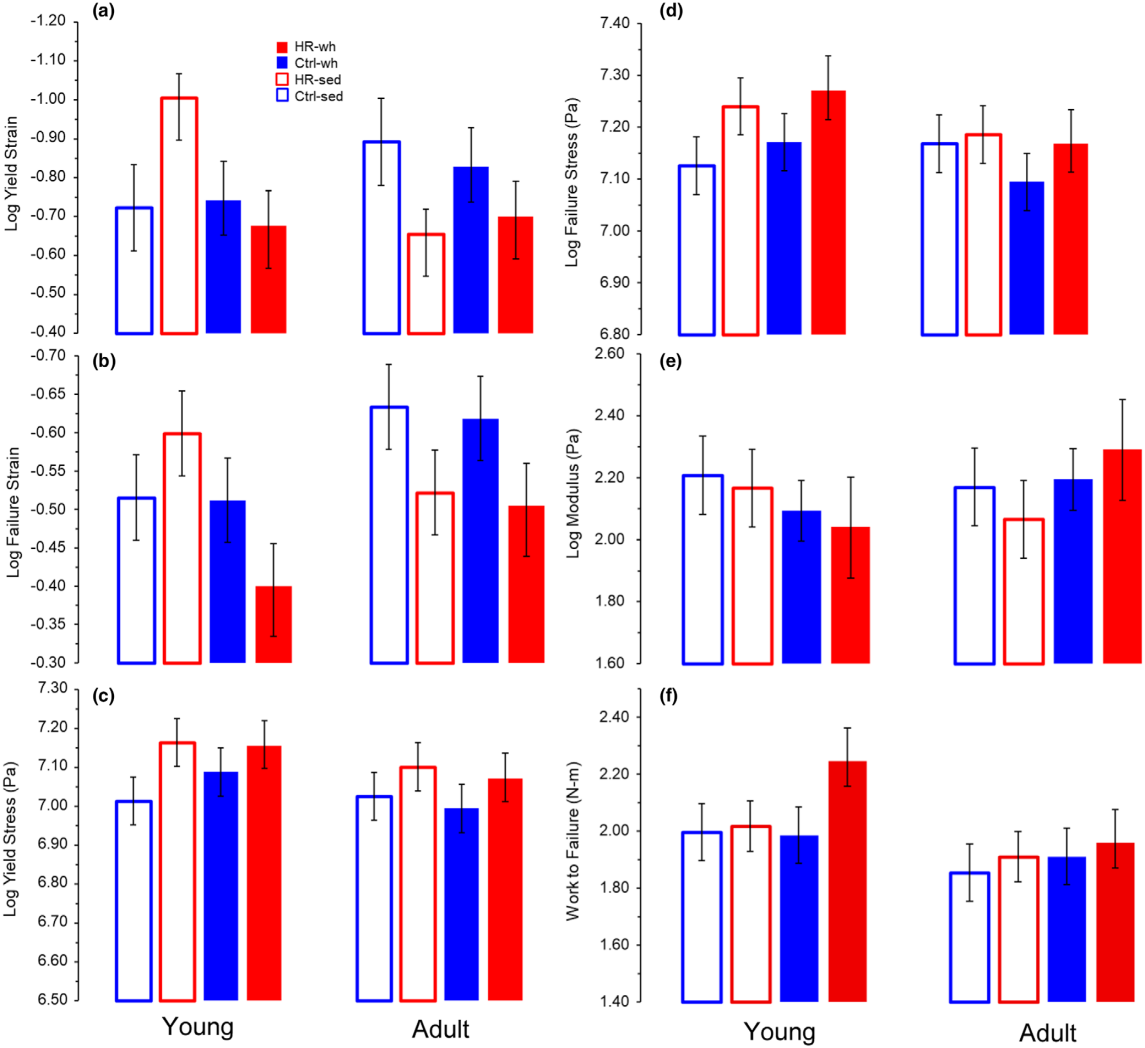
Least squares means (LSM) for tendon material properties taken from HR and Control mice after the training period beginning at either young (3 weeks old, left side of graphs) or young adult (9 weeks old, right side of graphs) stages. Least squares means (LSM) of yield strain (a), yield stress (b), failure strain (c), failure stress (d), modulus (e), and work to failure (f) from mixed model analyses with Age, Exercise, Line as fixed effects, and body mass as a covariate. Control mice are indicated in blue, HR mice in red, and wheel exercised mice are indicated with a filled bar while sedentary cohorts have bars with no fill. Yield (a) and failure (b) strains tended to be shorter in older cohort HR mice, and within younger HR mice, wheel access mice had >30% shorter yield and failure strains than sedentary HR mice (Table 1, differences of LSM). Yield stress was significantly greater in HR mice (*p* = 0.0262), but only approached significance for failure stress (*p* = 0.0676). Likewise, modulus (e) appeared to have reverse effects of exercise depending on the age cohort, but this interaction did not reach the significance threshold (*p* = 0.0632). Work to failure (f) was significantly greater in the younger cohort overall (*p* = 0.0435).

Tendon mechanical properties were variably affected by line and age, and interactions, but not exercise alone (all variables log‐transformed; Figure 5, Table 1). Yield strain (Figure 5a, Table 1) was marginally affected by an interaction between line and age (*p* = 0.0535). Within younger HR mice, wheel access increased yield strain (*p* = 0.0445) but did not in the older cohort (differences of Least Squares Means in SAS Proc Mixed, Table 1 right‐most columns). Failure strain was not significantly impacted by main effects, but within HR mice was much shorter in tendons from younger mice with wheel access when compared to sedentary (*p* = 0.0246; Figure 5b, Table 1 LSM means differences). Yield stress (*p* = 0.0262) and somewhat failure stress (*p* = 0.0676) were higher in HR mice across cohorts (Figure 5c,d, Table 1). Modulus demonstrated complex interactions of age cohort and exercise (*p* = 0.0632), as well as within older HR mice between sedentary and exercised (*p* = 0.0794; Figure 5e, Table 1 LSM means differences). Otherwise, modulus was not significantly impacted by any main effect or interaction, nor by body mass (Figure 5e; Table 1). Work to failure, the energy absorbed by the tendon up to structural failure, was greater in younger mice (*p* = 0.0435) than in the older cohort (Table 1, Figure 5f).

## 4 DISCUSSION

The major aim of this study was to investigate the potentially interactive effects of exercise and age on tendon structure and function. More specifically, we sought to determine the effects of high levels of exercise (i.e., high runner (HR) vs. control line mice) on tendon properties, and whether voluntary wheel running exercise had differential impacts on tendon before and after skeletal maturity. Although a previous study found that HR mice have longer, thinner tendons (Castro et al., 2024), the HR mice only differed from control line mice after wheel access (line*exercise *p* = 0.0410). Exercised mice of any line tended to have shorter tendons, and this was significant within post‐skeletal maturity HR mice groups. As with previous studies (i.e., Castro et al., 2024), HR mice had significantly smaller gastrocnemius muscles (Table 1). Somewhat surprisingly, HR mice tendons had significantly higher yield stress and marginally higher failure stress (Figure 5c,d). Yield and failure strains were significantly impacted by exercise within the young HR cohort, such that younger HR sedentary mice had >30% longer yield and failure strains than wheel‐access HR mice (Figure 5a,b). Despite the end of the experiment corresponding to skeletally mature mice (11 weeks and 18 weeks, respectively), there were some indications that mice in the younger cohort were still undergoing maturation as body mass was smaller in the younger cohort. Exercise by age interactions would indicate differential impacts of exercise related to timing pre‐ and post‐puberty, but only modulus approached significance (*p* = 0.0632; Figure 5e). However, work to failure was significantly greater in the young cohort overall, particularly in the HR wheel access mice (Figure 5f). However, after post hoc corrections for multiple comparisons (see Results) these terms would not be considered statistically significant. We believe the biological relevance is worth further interpretation; however, we thus continue to discuss and interpret the findings further.

The lack of stronger responses in tendon morphology and materials properties is somewhat surprising, given that (1) HR mice ran considerably more than usual test subjects, averaging three times the distance that control mice ran each week (Figure 3), (2) that tendon is purported to be much more dynamic during early maturation across a wide range of taxa than at any other stage (Choi et al., 2020; Svensson et al., 2017), and (3) that exercise has previously been shown to have effects on tendon properties in a variety of organisms (Couppé et al., 2008; Curwin et al., 1988; Wood & Brooks, 2016). Below we discuss the ramifications and caveats of these findings, as well as further exploring the results within the HR line in isolation.

Previously reported exercise effects on tendon morphology are highly variable, in part due to differences in training protocol regimes (e.g., endurance vs. resistance, acute vs. long‐term) (Table S1). For example, cross‐sectional studies on humans suggest that habitual endurance and resistance training are associated with a larger tendon CSA (Couppé et al., 2008; Kongsgaard et al., 2005; Magnusson et al., 2003; Yu et al., 2022) with greater changes associated with resistance training (Arampatzis et al., 2007; Kongsgaard et al., 2007; Seynnes et al., 2009). However, acute training programs did not find changes in the Achilles tendon CSA in novice human subjects (Hansen et al., 2003). Results from nonhuman studies tend to vary considerably more, with some demonstrating no discernible effects including several rodent studies that reported no changes in tendon diameter or CSA in response to endurance treadmill training (Heinemeier et al., 2012; Huang et al., 2004; Wood & Brooks, 2016), voluntary wheel running (Legerlotz et al., 2007), or resistance training protocols (de Cássia Marqueti et al., 2017; Legerlotz et al., 2007). The observation in this study that 8 weeks of wheel running did not induce gross structural changes in the Achilles tendon of control or HR mice agrees with these results, but not with other studies that have demonstrated increased tendon mass and CSA following uphill treadmill running in mice (Mendias et al., 2012) or mechanical loading via synergistic ablation (Gumucio et al., 2014). Results from tendons in other animals suggest that tendon function and anatomic location influence adaptation to exercise (Buchanan & Marsh, 2001; Viidik et al., 1996; Woo et al., 1981). For example, Birch et al. (1999) demonstrated hypertrophy in extensor tendons in horses following high‐intensity exercise training, but not in flexor tendons. Studies have suggested that tendons hypertrophy in response to increased loading exercises only after repetitive use over long periods, but under certain conditions, there is an acute response. A clear understanding of the mechanisms underlying tendon growth following exercise remains elusive.

Similar to tendon morphology, there is a lack of consensus with regard to the effects of exercise on tendon mechanical properties. Previous research has demonstrated changes in tendon mechanical properties in response to exercise with or without changes to tendon morphology (Svensson et al., 2017; Wiesinger et al., 2015). However, results vary considerably across training regimes and species, making it difficult to predict the direction and magnitude of changes in specific tendon properties. For example, despite using a similar uphill running protocol, Wood and Brooks (2016) reported a decrease in the plantaris tendon stiffness and elastic modulus in mice, whereas Heinemeier et al. (2012) observed greater elastic modulus and failure stress in the Achilles tendon from rats. In addition, tendon remodels differently from endurance compared to resistance training. It is generally accepted that resistance training leads to increased tendon stiffness and elastic modulus due to increased loading; however, these findings are largely derived from human studies (Arampatzis et al., 2007; Seynnes et al., 2009) and not studies on rodents or other animals (de Cássia Marqueti et al., 2017; Legerlotz et al., 2007). Mechanical responses to endurance training are highly variable (Heinemeier et al., 2012; Legerlotz et al., 2007; Woo et al., 1981; Wood & Brooks, 2016) and potentially sensitive to exercise intensity (Simonsen et al., 1995; Sommer, 1987). While exercise alone did not produce main effects, HR tendons showed specific mechanical responses. Notably, in younger HR mice, exercise significantly reduced both yield and failure strain, suggesting greater mechanical sensitivity in this subgroup. Together, these findings support the idea that the Achilles tendon could be responsive to exercise intensity, which would help to prevent damage due to mechanical fatigue (Buchanan & Marsh, 2002; Smith et al., 2002).

Tendon morphology and mechanics also vary across ontogeny (Curwin et al., 1988; Svensson et al., 2017), and it has previously been suggested that tendon may be better able to remodel at earlier stages of growth than later (Smith et al., 2002). Although exercise and ontogeny have each been relatively well‐studied, the effects of loading history during tendon maturation still are not well understood. Tendon is a dynamic tissue during maturation, with high cellular synthesis activity, increasing fibril diameter, and increasing cross‐link formation (Moore & De Beaux, 1987; Nakagawa et al., 1996; Zs.‐Nagy et al., 1969). Concomitant with these changes is an increase in cross‐sectional area (CSA) and subsequent increase in Young's modulus, a ratio of tensile stress to strain. Unlike other musculoskeletal tissues, however, tendon may be limited in its ability to remodel after maturity due to a steep decrease in extracellular matrix turnover, among other factors. This constraint in tissue turnover rate in mature tendon contributes to its relatively slow healing time (Steiner, 1982) as well as a limited response to some forms of exercise loading. Multiple lines of evidence from human studies suggest that tendon “cores” may reach a relatively static state by as early as 14 years (Tumkur Anil Kumar et al., 2021) and no later than 17 years of age (Heinemeier et al., 2013). Similarly, data from mouse tendons show that tendons continue to grow until about 10 weeks of age (Michna, 1984), after which only the outer epitenon appears to experience significant cellular turnover (Gumucio et al., 2014). However, a more recent study in rats (Choi et al., 2020) found that protein turnover rates in tendon may be better understood in the context of the spatially distinct fascicular and interfascicular extracellular matrix phases of tendon. In rats, the relatively collagen‐rich fascicular matrix demonstrated turnover rates (>1000 days) orders of magnitude longer than the glycoprotein‐rich interfascicular matrix (1.5 days), suggesting remodeling may be driven from outside the tendon fascicles themselves. Taken together, the variable response of tendon to exercise may be in part explained by variation in tendon used, study species, scale of study, and ontogenetic timing of loading history.

### 4.1 Comparisons with other studies of the high runner mouse model

The present results add to our knowledge of the evolutionary adaptations that have resulted from long‐term selective breeding for high levels of voluntary wheel‐running behavior in the HR mice. Previous studies have demonstrated a number of characteristics that seem to represent physiological or morphological adaptations for long‐distance, aerobically supported running, including higher endurance and maximal oxygen consumption (VO_2_max) during forced treadmill running (Meek et al., 2009; Schwartz et al., 2023), larger heart ventricles (Schwartz & Garland, 2024), and changes in skeletal traits (Castro et al., 2021) and muscle contractile properties (Castro et al., 2024). Changes to the brain's motivation and reward systems have also been documented (Rhodes et al., 2005; Garland Jr, Schutz, et al., 2011; Keeney et al., 2012; Schmill et al., 2023; Khan et al., 2024). These adaptations represent characteristics that have evolved to differ statistically in comparisons of all 4 HR lines with all four of the nonselected Control lines.

In our present investigation, results suggest that HR mice have relatively stronger tendons, and that HR mouse tendons respond more strongly to exercise. Although control‐line mouse tendon length, yield strain, and failure stress (Figures 4, 5; Table 1) did not change to a statistically significant degree after 8 weeks of access to wheels, HR mice showed a more marked response. Tendon length was significantly affected by a line*exercise interaction (*p* = 0.0410), with HR sedentary mice having longer tendons than their wheel‐access counterparts, and this effect being stronger in the older HR cohort (*p* = 0.0282). Yield and failure strain were significantly shorter in exercise cohorts in younger HR mice (*p* = 0.0445 and *p* = 0.0246, respectively). Traditionally, increased tendon length is associated with increased endurance‐running performance, both within populations over proximate time (e.g., human endurance runners Ueno et al., 2018) and among species over evolutionary scales (Polly & Hall, 2007). Longer tendons allow higher strains within a stride cycle, which in turn allows for greater storage and recovery of elastic energy during running (Pollock & Shadwick, 1994; Roberts, 2002). Therefore, it is somewhat surprising that in the present study, exercised HR tendons were *shorter* than in sedentary mice. However, these findings require cautious interpretation due to (1) existing baseline differences in morphology between HR mice and control lines and, (2) timing of exercise exposure with maturation and growth. Previous studies report that HR mice as a whole (i.e., considering all four replicate lines) have several baseline morphological differences from control‐line mice, including longer, thinner limb tendons and smaller muscles, although the magnitude of these differences varies somewhat among the HR lines (Castro et al., 2022, 2024; Castro & Garland Jr, 2018). Additionally, in the present study, both the young (training period: weanling at 3 weeks through 11 weeks) and adult (training period: 9 weeks through 18 weeks) exercise cohorts experienced growth during training, albeit post‐adolescent growth in the “adult” cohort. Therefore, it is possible that the exercise exposure resulted in stunted or arrested growth overall, as has been reported in prepubertal athletes (Georgopoulos et al., 2004; Malina, 1994) and sometimes in females from both HR and control lines of mice (e.g., see Dumke et al., 2001; Kelly et al., 2017; Swallow et al., 1999), and that the effect in the present study was strongest in the HR mice due to their extreme running. Our results suggest that, unlike the tendon of mice from the control line that we studied, the tendons from this line of HR mice can change in response to chronic endurance running by developing a shorter tendon with a relatively longer yield and failure point.

Here, we used HR mice primarily to increase the amount of voluntary running and hence increase power to detect training effects, if they were to occur. Due to constraints on physical resources, in particular the number of data‐logging wheels available, we used only one representative HR line and one control line. This is important to note, because a number of differences among the replicate control or HR lines have been demonstrated, including the extent to which their increased daily running distance has evolved by increased duration versus average running (Garland Jr, Kelly, et al., 2011. In the HR lines, some of these differences, including those observed in muscle properties, appear to represent “multiple solutions” to the adaptive “problem” imposed by our artificial selection protocol (Castro et al., 2024; Garland Jr., 2003; Hillis et al., 2024). Hence, comparisons of one HR and control line as in this study should not be assumed to reveal evolutionary adaptations (as opposed to differences caused by random genetic drift in the two lines used) or the lack thereof. Strong inference about adaptation requires consideration of all eight lines in this system.

One general hypothesis derived from the present results (based on one HR and one control line) in isolation is that tendon properties may not be crucial for enabling high levels of daily running (at least over 6 days) in animals as small as mice, even when the prevailing directional selection pushes populations to limits that are arguably of evolutionary significance (Dewsbury, 1980; Garland Jr., 2003) and keeps them there for many generations. However, that hypothesis can be refuted by results from a recent study of all eight lines, in which mice from the HR lines had significantly longer Achilles tendons (and shorter muscle bellies) as compared with the four control lines (Castro et al., 2024). Moreover, our results here are consistent with the general pattern that the sedentary HR mice tended to have longer tendons than those from the control line (Figure 4c). Our results for the gastrocnemius muscle (Table 1) are also consistent with previous findings that HR lines have smaller triceps surae muscle masses (Castro et al., 2024).

### 4.2 Limitations and practical considerations

As noted above, our study was somewhat limited in its approach due to practical constraints, as well as the intended scope of investigation. First, we only used female mice, which differ from male mice in running (e.g., Bartling et al., 2017; Careau et al., 2013; Garland Jr, Kelly, et al., 2011, tendon response to exercise (Magnusson et al., 2007), and body size. Second, the exercise loading consisted of voluntary running on unrestricted mouse wheels, which may not have been intense enough to induce gross morphological and mechanical changes despite the high running frequency in HR mice. In other studies that utilized endurance exercise training, there were similar trends with small or no changes in tendon properties (e.g., humans Kongsgaard et al., 2005; rats, Legerlotz et al., 2007). In addition to an increased propensity for running, HR mice have a tendency for a more gracile phenotype than mice from the nonselected control lines, with smaller overall body mass, reduced muscle mass, and thinner cortical bone (Castro et al., 2021, 2024; Castro & Garland Jr, 2018; Wallace et al., 2012).

As previously mentioned, the mice in the adult cohort grew to larger sizes than the weanling mice, regardless of line or exercise treatment. Weanling (3 weeks old) mice exposed to wheel exercise for 8 weeks were sacrificed at 11 weeks of age, which has been reported as skeletally mature for adult mice (Beamer et al., 1996; Patton & Kaufman, 1995). However, our comparisons of the “young” cohort mice at 11 weeks old and the “adult” cohort mice at 18 weeks of age demonstrated clear differences in body mass (Table 1, Figure 4a). In fact, mice and other rodents that are not bred at maturity continue to grow (although an asymptote is reached ~10 weeks for most mouse strains) for the majority of their lifespan (Kilborn et al., 2002). We therefore were limited in our direct comparisons between the age cohorts even with body mass used as a covariate for all statistical tests, as there may have been other underlying differences in maturation of musculoskeletal tissues that were not known to us. Additionally, the Achilles tendon is a challenging tissue in which to study materials properties. The Achilles tendon is utilized widely for tendon materials testing due to its pivotal role in hindlimb propulsion, and its common experimental usage allows for comparisons across a diverse array of taxa. However, the underlying anatomical complexity (three separate muscle insertions, low length‐to‐diameter ratio) contributes to a phenomenon of overall heterogeneous strain (Doral et al., 2010; Rigozzi et al., 2009).

## 5 CONCLUSION

In conclusion, this study highlights the complexity and variability in tendon responses to exercise, especially when considering such factors as species, age, and training protocols (see also Table S1). After correction for multiple comparisons, our (quite conservative) statistical approach found no significant main effects of line, exercise exposure, or age at exercise exposure. However, some interactions were apparent, and our deeper investigation within the HR line showed that, due to genetic predisposition and/or extreme running, older exercised HR tendons were shorter, and within the younger HR cohort, both yield and failure strain were shorter with exercise exposure. Future research should investigate the longer‐term protective effects of exercise while also addressing the lack of consensus due to variations in the muscle‐tendon unit (MTU), species, sex, maturation stage, training protocols, and methodologies. Even among rodent models, differences in tendon structure and function present challenges in generalizing results across species. Additionally, further clarification is needed to determine which specific cells and regions contribute to tendon remodeling post‐maturation, as this may significantly impact how tendons adapt to exercise over time.

## ETHICS STATEMENT

All animal experiments were approved by the Institutional Animal Care and Use Committee of California State University San Bernardino and were carried out under the guidelines of the National Institutes of Health Guide for the Care and Use of Laboratory Animals.

## Supporting information


**Table S1.**Literature values of aging and exercise effects on tendon materials properties. Methodological approaches for 29 studies were parsed by 1) subject age, exercise 2) type & 3) duration, and 4) tendons used. The experimental effects were noted for the tendon cross‐sectional area (CSA) and modulus to show the variation in scientific findings. Personal observations about the studies were also noted.
