# Supplementary material for: Early‐life exercise effects on Achilles tendon in mice selectively bred for high voluntary wheel‐running behavior
Source: Physiol Rep. 2025 Aug 19;13(16):e70515. doi: 10.14814/phy2.70515 (PMC12361812; doi:10.14814/phy2.70515)
Supplement: Supplementary file 1 — Table S1.Literature values of aging and exercise effects on tendon materials properties. Methodological approaches for 29 studies were parsed by 1) subject age, exercise 2) type & 3) duration, and 4) tendons used. The experimental effects were noted for the tendon cross‐sectional area (CSA) and modulus to show the variation in scientific findings. Personal observations about the studies were also noted. [file PHY2-13-e70515-s001.zip › Supplemental Table 1.docx]

Supplemental Table 1. Literature values of aging and exercise effects on tendon materials properties. Methodological approaches for 29 studies were parsed by 1) subject age, exercise 2) type & 3) duration, and 4) tendons used. The experimental effects were noted for the tendon cross-sectional area (CSA) and modulus to show the variation in scientific findings. Personal observations about the studies were also noted.
